# Evaluation of peritoneal Carcinoembryonic Antigen as a survival prognostic factor in gastric cancer patients: a single western center experience

**DOI:** 10.1007/s13304-023-01542-3

**Published:** 2023-06-22

**Authors:** Nicola Natalizi, Elisabetta Marino, Luigina Graziosi, Annibale Donini

**Affiliations:** General and Emergency Surgery, Santa Maria della Misericordia Hospital–University of Perugia, Via dottori, 06132 Perugia, Italy

**Keywords:** Gastric cancer, Peritoneal washing, Carcinoembryonic Antigen

## Abstract

The aim of this study is to define the importance of peritoneal CEA (pCEA) as a prognostic factor of overall survival (OS) and disease-free survival (DFS) in gastric cancer (GC) patients surgically treated with a curative intent In our department. A total of 64 patients affected by gastric cancer with intraoperatively measurement of CEA on peritoneal lavage were enrolled in the study. Patients were divided into two groups: (A) the peritoneal lavage CEA ( −) with CEA < 0.5 ng/ml and (B) the peritoneal lavage CEA ( +) with CEA ≥ 0.5 ng/ml. Then we analyzed OS and DFS of the two groups correlating them to others clinico-pathological features. Furthermore, we investigated the correlation between pCEA and peritoneal cytology. We demonstrated a strong significant difference in OS and in DFS in CEA ( +) patients. We emphasized that pCEA had a strong survival impact, in both OS and DFS, in selected patients affected by diffuse histotype GC (*p* = 0.0048 and *p* = 0.0030 respectively), stage III (*p* = 0.015 and *p* = 0.021, respectively) and distal gastric cancer (*p* = 0.0036 and *p* = 0.0017, respectively). There is a strong need to recognize prognostic factors that can help clinicians to stratify patients at high risk to develop post-surgical recurrences and moreover to recognize who could benefit from an aggressive surgical treatment of cytoreductive surgery and intra-peritoneal chemotherapy.

pCEA is a good predictor of survival in advanced gastric cancer and could discriminate which patients need a more accurate follow-up program and an intensive therapeutic strategy.

## Introduction

Gastric Cancer (GC) remains worldwide the most common cause of death for neoplasia although its incidence has been decreased during last years [1].

Showing such a bad prognosis, there is a strong need to individuate prognostic factors that could help clinicians to recognize the patient at high risk of recurrences after curative surgery and to identify the right therapeutic strategy and the correct follow-up.

Over the time, peritoneal cytology (PCY) has became a useful factor to predict individual prognosis in some gastrointestinal malignancies [2].

Especially in gastric and pancreatic cancers, the presence of free peritoneal malignant cells is considered a negative prognostic factor being associated with poor survival and peritoneal recurrences [3], since positive PCY was inserted in GC TNM staging system.

The presence of intra-peritoneal free malignant cells could be certainly detected by peritoneal cytology evaluation [3, 4] but it could be also detected by the intra-peritoneal Carcinoembryonic Antigen (pCEA) level assessment.

During the years, many authors have demonstrated that an high pCEA value showed a strong correlation with post-surgical local and peritoneal recurrences as Kim JH demonstrated in his work in which pCEA is considered an interesting predictive marker of survival in particular in I–III stages colorectal cancer patients when peritoneal cytology results negative [4].

To date, there are not so many works describing the role of pCEA in GC resected patients. For this reason we would like to evaluate the prognostic role of intra-peritoneal CEA and its association with gastric cancer peritoneal or extra peritoneal post-surgical recurrences.

## Materials and methods

We included 64 patients who underwent curative surgery for gastric adenocarcinoma from January 2014 to July 2017 treated in the department of "General and Emergency Surgery” of “Santa Maria della Misericordia Hospital” in Perugia.

We excluded patients who underwent surgery for linfoma, GIST or other gastric neoplasia, Sievert I and II carcinomas, patients who underwent palliative surgery, patients < 18 years old and patients with incomplete data.

Each patient was discussed at the multidisciplinary meeting before any surgical and medical treatment.

Locally advanced GC patients underwent neoadjuvant treatment as guidelines recommended. Chemotherapy regimens with DOX, FLOT or FOLFOX were generally administrated.

CEA and cytology were taken during the surgical act before tumor manipulation from ascitic fluid (if present) or after peritoneal lavage made with 100 ml of saline solution 0.9% in the supra and sub-mesocolic space.

This fluid was collected and then analyzed to identify gastric cancer intra-peritoneal free cells by the pathologist utilizing Papanicolau examination. An intra-peritoneal liquid sample was also analyzed to quantify the level of pCEA with ELISA technique.

We considered as pCEA cut-off the value of 0.5 ng/ml as reported in the literature [5, 6].

Furthermore, CEA was evaluated upon the admission of the patient from blood sampling.

Pre-operative biochemical, clinical and pathological data of each patient were inserted in a prospectively collected database and retrospectively analyzed as approved by the local University ethical committee. All the patients sign a written consent to be included.

After surgery all specimens were examined according to the 8th American Joint Committee on Cancer/Union for International Cancer Control (TNM).

All patients were followed up with physical examination, laboratory tests and imaging every 3–6 months in the first year and then every 6–12 months for at least 5 years after surgery.

### Statistical analysis

Continuous variables are presented as the mean ± standard deviation or median, and categorical variables are presented as frequencies and percentages. The differences between clinico-pathological characteristics were compared using chi-square test when applicable. Survival curves and disease survival rates were determined using the Kaplan–Meier method, and differences were compared using the log-rank test. Data management and statistical analyses were performed using SPSS software (SPSS 20.0, Chicago, IL, USA) and Prism 9 software. *P* < 0.05 was considered statistically significant.

## Results

Patients’ clinical pathological characteristics were analyzed. Median age was 74 and male female ratio was 2.76. Stage distribution of the entire population was as follow: stage I 23.4%, stage II 23.4%, stage III 23.4% and 29.7% stage IV.

15.63% of the entire population underwent neoadjuvant chemotherapy.

Subdividing patients according to peritoneal CEA we obtained two groups of 33 and 31 patients with negative and positive peritoneal CEA, respectively.

The two groups were homogeneous as there were non-significant factors differing between them, except for the stage, as shown in Table 1.

**Table 1:** Clinico-pathological features

	pCEA + (%) *n* = 31	pCEA- (%) *n* = 33	*p*-value
*Sex*			
Male	25 (80.65)	22 (66.67)	0.326
Female	6 (19.35)	11 (33.33)	
*Age*			
< 65	8 (25.81)	7 (21.21)	0.889
≥ 65	23 (74.19)	26 (78.79)	
*Localization*			
Fundus	4 (12.90)	2 (6.06)	0.307
Body	12 (38.71)	9 (27.27)	
Antrum	15 (48.39)	22 (66.67)	
*Histotype*			
Diffuse	9 (29.03)	9 (27.27)	0.976
Mixed	5 (16.13)	5 (15.15)	
Intestinal	17 (54.34)	19 (57.58)	
*pT*			
1	2 (6.45)	8 (24.24)	< 0.05
2	2 (6.45)	8 (24.24)	
3	13 (41.94)	10 (30.31)	
4a	13 (41.94)	6 (18.18)	
4b	1 (3.22)	1 (3.03)	
*pN*			
0	8 (25.81)	17 (51.51)	< 0.05
1	3 (9.68)	3 (9.09)	
2	4 (12.90)	9 (27.27)	
3a	4 (12.90)	4 (12.13)	
3b	12 (38.71)	0 (0.00)	
*Lymphadenectomy*			
D1	3 (9.68)	7 (21.21)	0.36
D2	17 (54.84)	18 (54.55)	
D3	11 (35.48)	8 (24.24)	
*Stage*			
I	3 (9.68)	12 (36.37)	< 0.05
II	6 (19.35)	9 (27.27)	
III	9 (29.03)	6 (18.18)	
IV	13 (41.94)	6 (18.18)	
*Peritoneal cytology (PCY)*			
Positive	13 (41.94)	7 (21.21)	0.069
Negative	18 (58.06)	23 (69.70)	
Not performed	0	3 (9.09)	
*Recurrence*			
Yes	17 (54.84)	11 (33.33)	0.139
No	14 (45.16)	22 (66.67)	
*Complications (Clavien–Dindo)*			
0	10 (32.26)	14 (42.42)	0.488
1	5 (16.13)	8 (24.24)	
2	12 (38.71)	8 (24.24)	
3a	1 (3.22)	2 (6.07)	
3b	0 (0.00)	0 (0.00)	
4	3 (9.68)	1 (3.03)	
5	0 (0.00)	0 (0.00)	
*Charlson Comorbidity Index*			
2	1 (3.22)	1 (3.03)	0.772
3	3 (9.68)	2 (6.07)	
4	4 (12.90)	5 (15.15)	
5	10 (32.26)	8 (24.24)	
6	6 (19.36)	4 (12.12)	
7	3 (9.68)	8 (24.24)	
8	3 (9.68)	4 (12.12)	
9	0 (0.00)	1 (3.03)	
12	1 (3.22)	0 (0.00)	
*Staging laparoscopy*			
Yes	5 (16.13)	4 (12.12)	0.919
No	26 (83.87)	29 (87.88)	

**Fig. 1:**
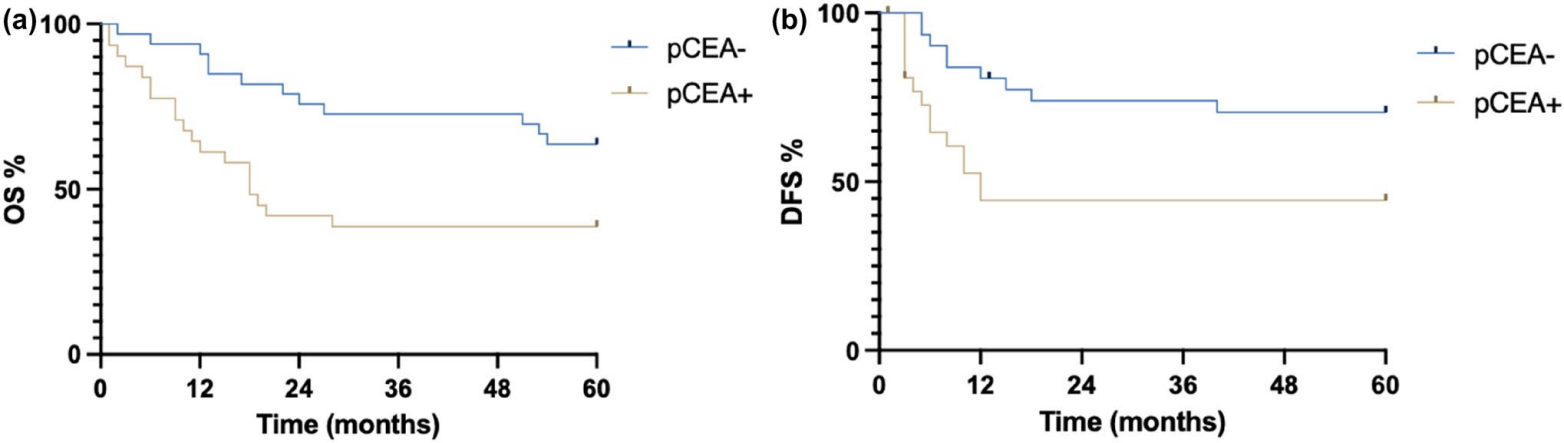
Global **a** OS; **b** DFS

We, therefore, analyzed overall survival showing that patients with positive peritoneal CEA had a worse prognosis both in terms of overall survival and in terms of disease-free survival as shown in Fig. 1a and b.

5-year overall survival of patients with positive CEA was 38% vs 64%, and 5-year DFS was 44% vs 70%.

Also subdividing patients according to the pathological stage a statistical significance was detected in stage III, but not in stages I, II and IV. This could be quite impacting on everyday clinical practice.

**Fig. 2:**
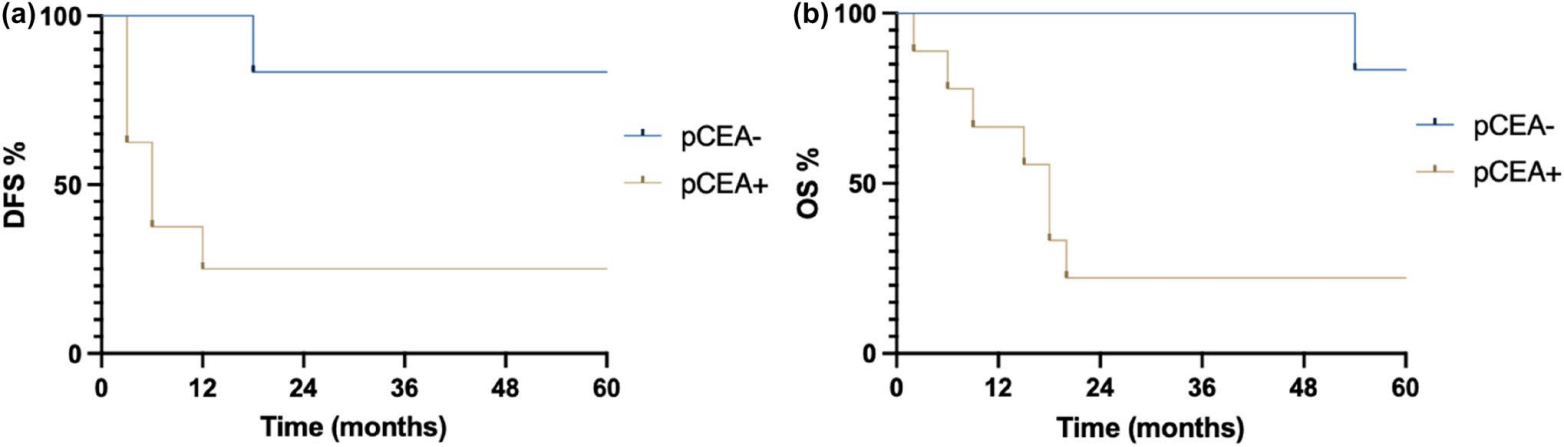
Stage III **a** 5Y DFS; **b** 5Y OS

The prognostic impact of positive pCEA in stage III both in overall survival (22% vs 83%) and in the disease-free survival (25% vs 83%) could be quite relevant in leading therapeutic approaches in those patients as represented in Fig. 2a and b.

Instead, in stage II, statistical significance is not reached in a statistically way but there is still a trend; patients with positive pCEA had a shorter survival than those with negative pCEA.

**Fig. 3:**
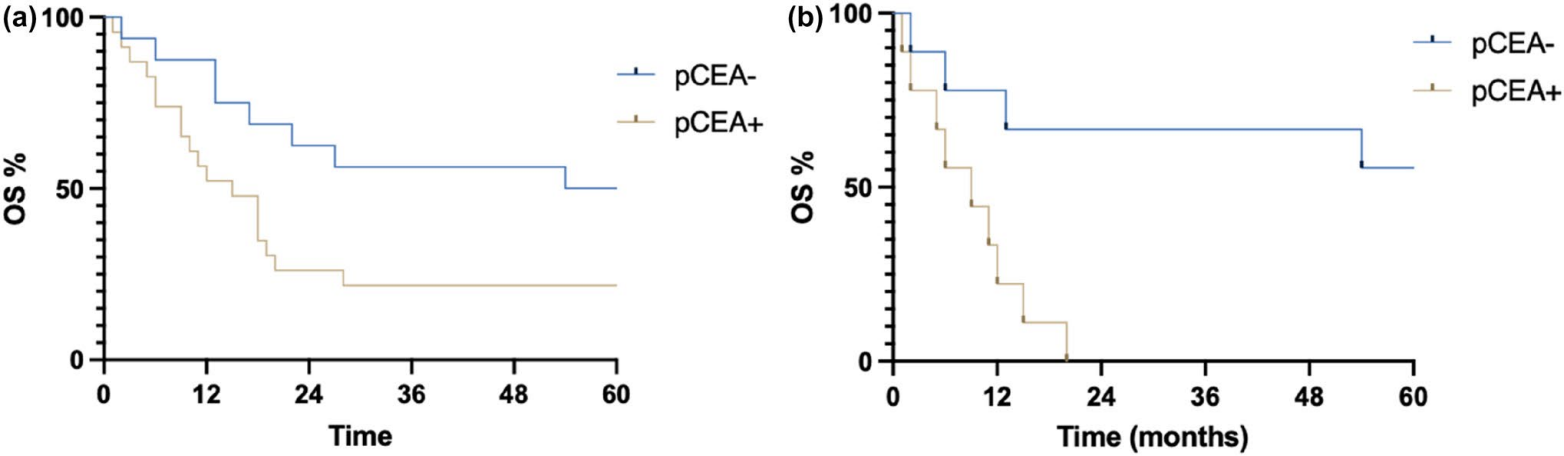
**a** N + ; **b** diffuse histotype

In addition, pCEA level negatively reinforces prognostic survival effect caused by lymph node involvement and diffuse histotype as shown in Fig. 3a and b.

**Fig. 4:**
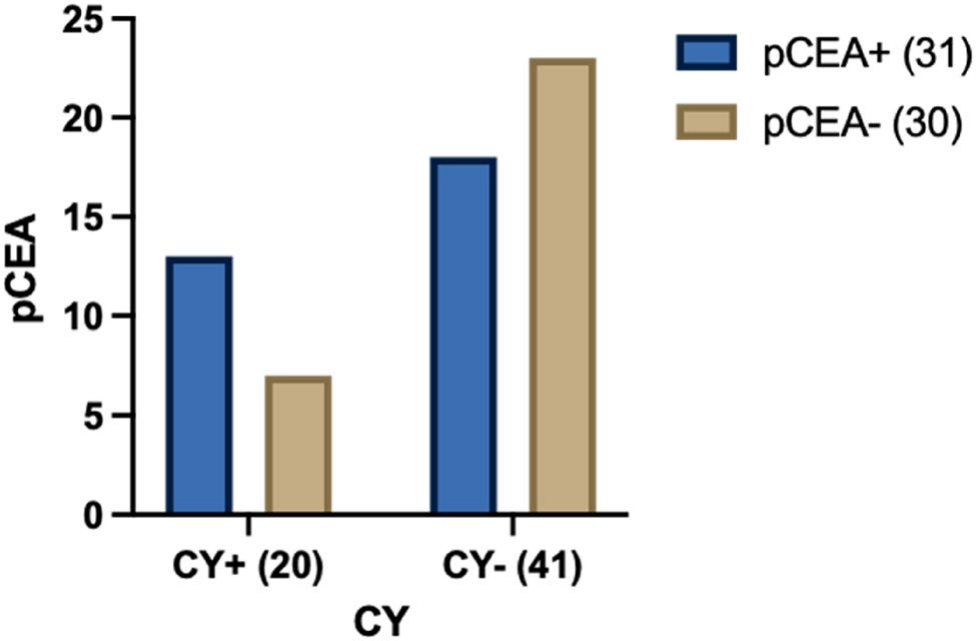
Contingency pCEA and CY

Lastly, we demonstrate a significant correlation between positive peritoneal cytology and positive pCEA as shown in Fig. 4.

At the multivariate analysis, pCEA is not an independent prognostic factor for survival.

**Fig. 5:**
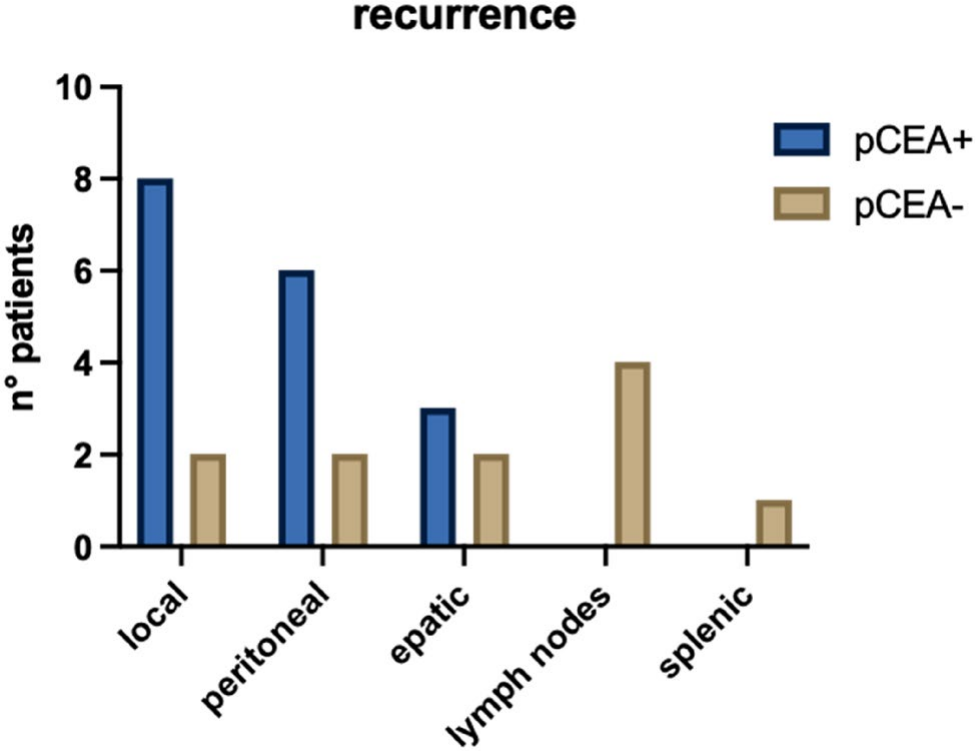
Correlation between pCEA and site of recurrence

We also evaluated the correlation between the pCEA and the pattern of recurrences; we estimated that patients with positive pCEA developed more local and peritoneal recurrences while those with negative pCEA more at the lymph node level (Fig. 5).

## Discussion

GC is the fifth most common malignant cancer and the third leading cause of cancer death in the world [1].

Among post-surgical recurrences, peritoneal involvement is the most common although a macroscopically curative surgery has been made.

This fact is caused by the presence of free malignant cells in the peritoneum that could be early detected by cytology evaluation. On the other hand, it is reported that more than half of positive cases were missed due to the limitations in the aspect of sensitivity and accuracy of the cytological examination.

Besides peritoneal cytology, CEA could be probably chosen as a good candidate marker for immunochemistry examination to evaluate the presence of free peritoneal malignant GC cells [7–10].

CEA is a glycoprotein normally produced in gastrointestinal tissue during fetal development, and the production of CEA decreases after birth [11]. Therefore, the level of CEA is very low in the blood of healthy adults and it remains an useful and common tumor marker for gastrointestinal cancer. This marker is easily and routinely measured without technical problem in comparison to the cytology determination.

Thus, we aimed to evaluate the predictive role of the carcinoembryonic antigen (CEA) intraoperatively detected inside the peritoneal lavage of GC patients who underwent curative surgery.

Several studies have reported that the assessment of CEA in intra-peritoneal fluid (pCEA) could have a diagnostic and prognostic value in particular in patients affected by colon cancer (CC) [12].

Kanellos et al. in 2006 demonstrated that the combination of positive cytology and high pCEA level revealed a high accuracy of 85% in predicting colon cancer local recurrence [13]. *Kim *et al. showed that high pCEA remained a strong risk factor of poor survival in resected colon cancer patient [14].

Concerning the prognostic correlation between GC and the pCEA, the literature is not so rich and the only existing studies were made only by eastern centers showing unconclusive results.

Qu L. et al. demonstrated that pCEA evaluation associated with cytology could better identify the GC patient in progression after surgery [15].

Also *Irinoda* showed that prognosis in patients with high pCEA level was significantly poorer than in those without it [16].

In our small series, we demonstrated a strong correlation between pCEA positivity and poor prognosis in terms of OS and DFS. pCEA positivity is associated with advanced stages of disease and it is more frequent in the population of CY + compared to CY- (59.0% vs 40.5%).

In addition, high pCEA level reinforced the negative survival impact caused by lymph node involvement, diffuse histotype according to Lauren classification [17] and advanced stage of disease.

In stage III, pCEA is a strong prognostic survival factor and it could be considered a valid instrument to identify the patient who needs an intensive strategy of treatment or a strict post-surgical follow-up.

Our study has some bias as the retrospective nature and the small size of the studied population. Therefore, we included patients who underwent neoadjuvant therapy that could falsify the results.

Our intention is to enlarge our results and to subsequently validate them in other surgical western centers.

## Conclusion

Concluding high pCEA level is correlated with poorer overall survival as well as recurrence-free survival.

These findings suggest that pCEA evaluation may has prognostic value for gastric cancer-resected patients and help clinicians in decision-making.

## Data Availability

Data will be available upon a motivated request to the corresponding author.
